# Number of blastocysts biopsied as a predictive indicator to obtain at least one normal/balanced embryo following preimplantation genetic diagnosis with single nucleotide polymorphism microarray in translocation cases

**DOI:** 10.1007/s10815-016-0831-0

**Published:** 2016-11-07

**Authors:** Yi-zi Wang, Chen-hui Ding, Jing Wang, Yan-hong Zeng, Wen Zhou, Rong Li, Can-quan Zhou, Ming-Fen Deng, Yan-wen Xu

**Affiliations:** 1 Reproductive Medical Center, The First Affiliated Hospital of Sun Yat-sen University, (Floor 1, Building 6th) ZhongshanEr Road No. 58, Yuexiu District, Guangzhou, Guangdong 510080 China; 2 Guangdong Provincial Key Laboratory of Reproductive Medicine, Guangzhou, China

**Keywords:** Robertsonian translocation, Reciprocal translocation, Preimplantation genetic diagnosis, Single-nucleotide polymorphism, Trophectoderm biopsy

## Abstract

**Purpose:**

The aim of this study is to investigate the minimum number of blastocysts for biopsy to increase the likelihood of obtaining at least one normal/balanced embryo in preimplantation genetic diagnosis (PGD) for translocation carriers.

**Methods:**

This blinded retrospective study included 55 PGD cycles for Robertsonian translocation (RT) and 181 cycles for reciprocal translocation (rcp) to indicate when only one of the couples carried a translocation. Single-nucleotide polymorphism microarray after trophectoderm biopsy was performed.

**Results:**

Reliable results were obtained for 355/379 (93.7 %) biopsied blastocysts in RT group and 986/1053 (93.6 %) in rcp group. Mean numbers of biopsied embryos per patient, normal/balanced embryos per patient, and mean normal/balanced embryo rate per patient were 7.4, 3.1, and 40.7 % in RT group and 8.0, 2.1, and 27.3 %, respectively, in rcp group. In a regression model, three factors significantly affected the number of genetically transferrable embryos: number of biopsied embryos (*P* = 0.001), basal FSH level (*P* = 0.040), and maternal age (*P* = 0.027). ROC analysis with a cutoff of 1.5 was calculated for the number of biopsied embryos required to obtain at least one normal/balanced embryo for RT carriers. For rcp carriers, the cutoff was 3.5. The clinical pregnancy rate per embryo transfer was 44.2 and 42.6 % in RT and rcp groups (*P =* 0.836).

**Conclusions:**

The minimum numbers of blastocysts to obtain at least one normal/balanced embryo for RT and rcp were 2 and 4 under the conditions of female age < 37 years with a basal FSH level < 11.4 IU/L.

## Introduction

Translocation is one of the most common structural rearrangements of chromosomes, with an estimated 0.4 and 0.19 % prevalence in prenatal samples and newborns [1]. Robertsonian translocation (RT) and reciprocal translocation (rcp) usually result in no obvious phenotypic abnormalities when balanced. However, both types of translocation carriers have a high rate of producing a chromosomally unbalanced gamete, which results in a high risk for miscarriage. For a RT carrier, depending on the segregation patterns in meiotic divisions, only 1/6 normal and 1/6 balanced gametes are alternately segregated, which can produce normal or balanced (normal/balanced) embryos [2]. For rcp carriers, the alternate gametes merely account for 1/9 [3]. The risk of miscarriage is estimated at 20–33 % in couples who carry RT and 47–53 % in couples who carry rcp [4].

Preimplantation genetic diagnosis (PGD) is used as an alternative for carriers of balanced translocation to select normal/balanced embryos for transfer to minimize the risk of miscarriage due to abnormal segregation of the translocation and to maximize the chance of a healthy child. Recently, a variety of biopsy methods combined with different biopsy stages and modes of embryo transfer have been attempted by PGD. In the PGD field, multiple displacement amplification (MDA)-based single-nucleotide polymorphism (SNP) arrays have been commonly utilized to discriminate euploid embryos from aneuploid embryos [5]. More recently, several polymerase chain reaction-based next-generation sequencing protocols have been developed and validated [6–8]. Both platforms can detect expected unbalanced segments arising from translocation chromosomes and distinguish aneuploidies from other chromosomes. In our center, SNP arrays are routinely used. In addition, blastocyst biopsy rather than cleavage-stage biopsy is now generally recommended because it has the advantage of a lower mosaic rate, reduced biopsy-related damage, and higher developmental potential [9].

With the advancement of diagnostic technology, PGD results for translocations have become more accurate. However, not every PGD patient has an adequate number of blastocysts for biopsy, and some patients may not have normal/balanced embryo for transfer even though they have several blastocysts for PGD. The multi-center analysis from the European Society of Human Reproduction and Embryology (ESHRE) consortium reported that only 26 % of successfully diagnosed embryos biopsied from translocation carriers were suitable for transfer [10]. The normal/balanced embryo rate (transferrable rate) for a RT male carrier, RT female carrier, rcp male carrier, and rcp female carrier were 38, 29, 22, and 20 %, respectively [11]. However, when the number of transferrable embryos is estimated according to the number of SNP array-biopsied blastocysts, or when determining the minimum number of blastocysts required by biopsy to obtain at least one normal/balanced embryo for carriers, these general data have limited values. The aim of this study was to investigate the minimum number of blastocysts required for PGD using a SNP array to maximize the chance of obtaining at least one normal/balanced blastocyst for transfer.

## Materials and methods

### Patient information

In this study, 51 patients with an indication of RT underwent 55 cycles of PGD and 131 couples with an indication of rcp underwent 181 cycles of PGD from July 2013 to December 2014. Translocation status was confirmed by conventional karyotype analysis. There were 16 couples involving female carriers and 35 couples involving male carriers in the RT group, and 67 couples involving female carriers and 64 couples involving male carriers in the rcp group. No compound translocation cases were recruited in either group.

### Ethical considerations

Written informed consent describing the limitations, advantages, and necessities of PGD was obtained from participants before couples underwent controlled ovarian stimulation (COS) cycles. All materials and diagnosis protocols in this study were approved by the Faculty of Medical Research Service Ethics Committee, the First Affiliated Hospital of Sun Yat-sen University, China.

### Controlled ovarian stimulation and in vitro fertilization

To obtain an ideal number of oocytes, flexible gonadotropin-releasing hormone (GnRH) agonist long protocols were performed. The starting doses of recombinant follicle-stimulating hormone (FSH) (Gonal-F, Merck-Serono, Geneva, Switzerland; Puregon, NV Organon, Oss, The Netherlands) and/or human menopausal gonadotrophins (HMG, Lizhu, China) were determined experientially according to patient age, body mass index (BMI), basal FSH, antral follicle count (AFC), and/or previous response to ovarian stimulation. According to the patient’s ovarian response, monitored by regular transvaginal ultrasound and plasma estradiol levels when necessary, the dose of recombinant FSH and/or HMG was adjusted individually. Oocyte retrieval (OR) through vaginal puncture under ultrasound guidance was performed 34–36 h after the administration of human chorionic gonadotropin (250 μg Ovidrel^R^; EMD-Serono). Only meiosis II (MII) stage oocytes were microinjected by ICSI 4–6 h after OR. A fertilization check was performed 16–18 h post-ICSI.

Three females in the RT group and 44 females in the rcp group received multiple controlled ovarian stimulations to accumulate a pool of embryos for testing. The number of stimulation cycles ranged from one to four.

### Embryo culture and biopsy

All embryos were cultured in sequential media (G1 and G2, Vitrolife, Goteborg, Sweden) to blastocyst stage under 6 % CO_2_, 5 % O_2_, and 89 % N_2_ in a COOK mini-incubator (Bloomington, IN, USA) for further manipulation. An 18–20-mm hole was then made by laser drilling in the zona pellucida of all embryos with no sign of hatching on the morning of day 5 (D5). Blastocysts with trophectoderm (TE) cells herniating out of the zona pellucida were chosen for biopsy on D5 or Day 6 (D6). Approximately 5–10 TE cells were aspirated with a biopsy pipette (internal diameter 30 μm) and dissected with an OctaxShot™ laser system. Biopsied TE cells were washed three times in G-MOPS medium (Vitrolife) and then either used directly for whole genome amplification (WGA) or stored at −20 °C for future WGA.

### Blastocyst vitrification and thawing

Blastocysts were vitrified after biopsy using a Kitazato vitrification kit (KitazatoBiopharma Co. Ltd., Shizuoka, Japan) in combination with Vitrification Cryotop. Each blastocyst was stored in an individual straw. The vitrification and thawing procedure was carried out according to the protocol recommended by the Kitazato vitrification kit.

### SNP microarray analysis

All biopsied TE cells were placed in 5 μl of DNA stabilizing buffer (0.2 M KOH) for SNP microarrays. The cell samples from each embryo first underwent cell lysis and the WGA protocol. For SNP arrays, cells were lysed using an alkaline denaturation buffer (0.2 M NaOH) followed by a 4-h modified MDA protocol using φ29 polymerase to generate template DNA. Then, 4 μL (200 ng) of DNA product was used in a 13-hour WGA amplification protocol using φ29 polymerase. Each DNA product then underwent enzymatic end-point fragmentation and the resuspended DNA samples were then dispensed onto Human CytoSNP-12 DNA analysis bead chips (Illumnia, San Diego, CA) and allowed to hybridize for 12 h. Each CytoSNP-12 bead chip contained approximately 301,000 SNPs and other genetic markers. Stringency washes were performed to remove un-hybridized and non-specific bound DNA. Bead chips dried in a desiccator were scanned using an Illumina iScan Bead Array Reader. Raw data analysis was accomplished using Illumina Genome Studio software. One of the limitations of the SNP array is the inability to distinguish a balanced embryo from a normal embryo.

### Embryo transfer and luteal support

Only blastocysts with concordant normal/balanced results were considered for embryo transfer. According to the patients’ menstruation characteristics, the natural ovulation cycle (NC) or hormone replacement treatment protocol (HRT) was used for endometrial preparation. Six days after observing a surge in serum luteinizing hormone or progesterone administration, qualified blastocysts, prioritized based on the best quality before biopsy, were thawed. Re-expanded blastocysts were transferred 3–4 h after warming. Individual luteal support protocols were administered. No more than two blastocysts were transferred.

### Outcome parameters

All parameters were classified according to the type of translocation. Patient baseline characteristics and COS parameter are presented in Table 1. Transferrable rate was calculated according to the equation: number of normal/balanced embryos/number of embryos biopsied and was the main PGD outcome. Biopsy outcomes of SNP arrays are summarized in Table 2. Pregnancy was confirmed 12 days after embryo transfer (ET) if the serum HCG was elevated. Clinical pregnancy was defined as the presence of fetal heartbeat (FHB) in the uterus under transvaginal ultrasound scan.

**Table 1:** COS parameters and results

	Robertsonian translocation	Reciprocal translocation
Couples	51	131
Female carriers	16	67
Male carriers	35	64
COS cycle	55	181
Patients with one stimulation cycle	48	86
Patients with ≥ 2 stimulation cycles	3	45
Average maternal age (years)	31.2 ± 3.7	30.7 ± 3.9
Maternal BMI (kg/m^2^)	20.8 ± 3.0	21.2 ± 4.1
Basal FSH (IU/L)	5.7 ± 1.8	5.7 ± 1.7
Basal E_2_ (pg/ml)	34.4 ± 15.8	36.0 ± 16.1
Initial dose of Gn (IU)	196.2 ± 61.4	198.9 ± 56.6
Stimulation length (days)	11.2 ± 1.7	11.2 ± 2.0
Total amount of Gn (IU)	2337.3 ± 913.8	2396.7 ± 878.4
HCG day E_2_ (pg/ml)	3207 ± 1429.5	2891 ± 1403.8
Retrieved oocytes/OPU	19.8 ± 8.7*	17.0 ± 8.2*
MII	16.4 ± 7.1*	14.0 ± 6.6*
2PN (fertilized)	12.6 ± 5.7	11.6 ± 5.6
Cleavage rate/%	94.8 ± 8.3	96.5 ± 10.2
Mean number of embryos biopsied	7.4 ± 3.6	8.0 ± 3.5
Number of normal/balanced embryos	3.1 ± 2.2*	2.1 ± 1.6*
Couples without Normal/balanced embryos for FET	4	16

Data are expressed as mean ± SD. Data refer to analyzed cycles that were only those who were completed; cancelled cycles are not included in the analysis

*COH* controlled ovarian hyperstimulation, *Gn* gonadotropins, *OPU* oocyte pick-up, *MII* metaphase II, *FET* frozen embryo transfer

*P <0.05; significant difference between Robertsonian translocation group and reciprocal translocation group ( Welch *t* test with F test for variance performed as appropriate)

**Table 2:** Outcomes of biopsied blastocysts

Population biopsy results	Robertsonian Translocation	Reciprocal Translocation	Maternal age ≥37 years	Maternal age <37 years
Biopsied embryos	379	1053	85	1347
Embryos diagnosed	355(93.7)	986(93.6)	77(90.6)	1264(93.8)
Normal/balanced translocation without aneuploidy	160(45.1)^a^	283(28.7)^a^	17(22.1)^b^	426(33.7)^b^
Balanced, with other aneuploidy	105(29.6)^a^	182(18.5)^a^	23(29.9)^b^	258 (20.4)^b^
Unbalanced, without other aneuploidy	72(20.3)^a^	425(43.1)^a^	25(32.5)^b^	473(37.4)^b^
Unbalanced, with other aneuploidy	18(5.1)^a^	96(9.7)^a^	12(15.6)^b^	107(8.5)^b^
Embryos undiagnosed	24(6.3)	67(6.4)	8(9.4)	83(6.2)
Total abnormality	195(51.4)	703(66.8)	60(70.6)	838(62.2)
Mean diagnostic rate of normal/balanced translocation %^c^	40.7 ± 21.8(34.6–46.9)	27.3 ± 19.8(23.9–30.7)	–	–

Values are expressed as *n* (%) or mean ± standard deviation of the mean (95 % confidence interval)

^a^Significant differences (*P* < 0.05) within translocation type (chi-square test): Pearson = 79.63, *P* < 0.001

^b^Significant differences (*P* < 0.05) within maternal age (chi-square test): Pearson = 10.69, *P* = 0.014

^c^Student’s *t* test: t = 3.99, *P* < 0.001

### Statistical analysis

All statistical procedures were conducted with SPSS version 22.0 software (SPSS Inc., Chicago, IL, USA). A combination of the Student’s *t* test, F test for variance, and chi-square test were performed. To determine correlative factors, a linear regression model (Y = B_0_ + B_1_X_1_ + B_2_X_2_) was used to analyze the entire data pooled from the two groups. Different associated variables (or prognostic factors) such as age, BMI, basal FSH, basal E_2_ level, total amount of Gn, E_2_ level on HCG day, number of oocytes retrieved, and number of biopsied blastocysts were entered as independent variables and the number of genetically normal/balanced blastocysts after PGD was regarded as a dependent variable. ROC curves were calculated to determine the cutoff value for the number of biopsied blastocysts with the aim of obtaining at least one normal/balanced blastocyst. *P* values < 0.05 were considered significant.

## Results

Fifty-one patients underwent 55 PGD cycles for RT and 131 couples underwent 181 cycles of PGD for rcp. To increase the number of embryos for PGD due to low ovarian reserve or poor response, multiple ovarian stimulation treatments (2–4 cycles) were performed for 3 RT carriers and 45 rcp patients. There was no difference in the mean maternal age in the RT and rcp groups (31.2 ± 3.7 and 30.7 ± 3.9 years, respectively). Other factors closely associated with the number of biopsy blastocysts are shown in Table 1. There were more retrieved and matured oocytes in the RT group compared with the rcp group (*P* < 0.05); however, the number of normally fertilized embryos and cleavage rate were similar in the two groups. The number of normal/balanced blastocysts was significantly higher for RT carriers compared with rcp carriers (*P* < 0.05), although the number of blastocysts available for biopsy was the same.

The outcomes of biopsied blastocysts are shown in Table 2. MDA-based WGA was processed successfully for 1341 samples. Of these, 355 were from the RT group and 986 were from the rcp group. The failure or “no result” rate was similar between the two groups. Poor embryo quality was the main factor attributed to amplification failure.

Among the diagnosed blastocysts, 160 (42.2 %) were normal/balanced with no aneuploidy in non-translocated chromosomes in the RT group, 105 (27.7 %) were balanced but with aneuploidy of non-translocated chromosomes, 72 (19 %) were unbalanced only in the translocated chromosomes, and 18 (4.7 %) were unbalanced along with abnormalities in other chromosomes. For RT carriers, the mean detection rate of transferrable blastocysts was 40.7 % (95% confidence interval [CI], 34.6–46.9 %).

In the rcp group, 283 (26.9 %) diagnosed blastocysts were normal/balanced with no aneuploidy in non-translocated chromosomes, 182 (17.3 %) blastocysts were balanced with aneuploidy in non-translocated chromosomes, 425 (40.4 %) of diagnosed blastocysts were unbalanced only in translocated chromosomes, and 96 (9.1 %) embryos were unbalanced with abnormalities in other chromosomes. For rcp carriers, the mean detection rate of transferrable blastocysts was 27.3 % (95 %CI, 23.9–30.7 %).

The rates for unbalanced translocation with or without sporadic aneuploidy were significantly higher for reciprocal translocation carriers (*P* < 0.01). Therefore, a significantly higher rate of unbalanced translocation was detected in the rcp group compared with the RT group. This translated into a comparatively lower detection rate of transferrable blastocysts for rcp carriers compared with RT carriers. A comparison of the detection rates in the two groups was statistically significant (*P <* 0.01).

Data was merged from the two groups and a linear regression model was used to examine exploratory variables such as age, BMI, basal FSH, basal E_2_ level, total amount of Gn, E_2_ on HCG day, number of oocytes retrieved, and number of biopsied blastocysts to number of genetically normal/balanced blastocysts after PGD. We identified three significant factors that affected the number of genetically transferrable embryos in the adjusted model: number of biopsied embryos (coefficient: 0.489, 95 %CI 0.350–0.557, *P* = 0.001), basal FSH level (bFSH) (coefficient: 0.138, 95 %CI 0.071–0.225, *P* = 0.040), and maternal age (mAge) (coefficient: 0.160, 95 %CI 0.089–0.201, *P* = 0.027). According to mathematical calculations, this significant impact only existed if the inequality was satisfied: 0.16* mAge + 0.138* bFSH ≤ 7.5.

**Fig. 1:**
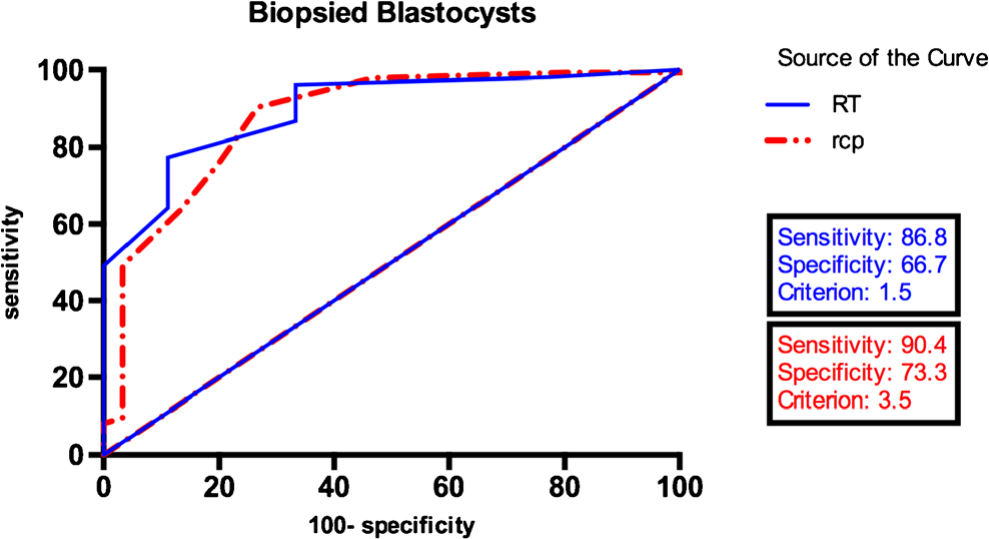
ROC curves for the relationship between the number of biopsied blastocysts and the outcome of obtaining at least one normal/balanced blastocyst in Robertsonian translocation and reciprocal translocation carriers in subgroups whose basal FSH levels were lower than 11.4 IU/L and females who were younger than 37 years of age

Because basal FSH levels were below 11.4 IU/L in all cases, a subgroup excluding females aged over 37 (4 couples in the RT group and 12 couples in the rcp group, 21 COS cycles totally) was created for further ROC analysis (Fig. 1). A positive result was defined as finding at least one normal/balanced blastocyst after biopsy. According to ROC curves, a threshold number of 1.5 biopsied blastocysts was identified in RT patients achieving positive results (sensitivity = 86.8 %, specificity = 66.7 %). For rcp carriers, the threshold number was 3.5 (sensitivity = 90.4 %, specificity = 73.3 %). The area under the curve (AUC) in the RT and rcp groups (0.912; 95 %CI: 0.812–0.998 and 0.877; 95 %CI: 0.801–0.953, respectively) indicated that it was a good prognostic test.

Single and double embryo transfers were performed for 139 translocation carriers (41 RT and 98 rcp carriers). Clinical pregnancy was achieved in 33 cases in the RT group and in 54 cases in the rcp group. The clinical pregnancy rate per ET was 44.2 and 42.6 % in the RT group and rcp groups, respectively (*P =* 0.836) (Table 3).

**Table 3:** Pregnancy outcomes in chromosomal translocation carriers

	Robertsonian translocation	Reciprocal translocation
Carriers with no ET	10	33
Carriers with ET	41	98
Cycles with ET	52	169
No. Embryo transferred	95	255
Clinical pregnancy rate per ET, %	44.2^N^	42.6^N^

^N^ No significant difference (*P* <0.05) between Robertsonian translocation group and reciprocal translocation group (chi-square test): Pearson = 0.043, *P* = 0.836

**Fig. 2:**
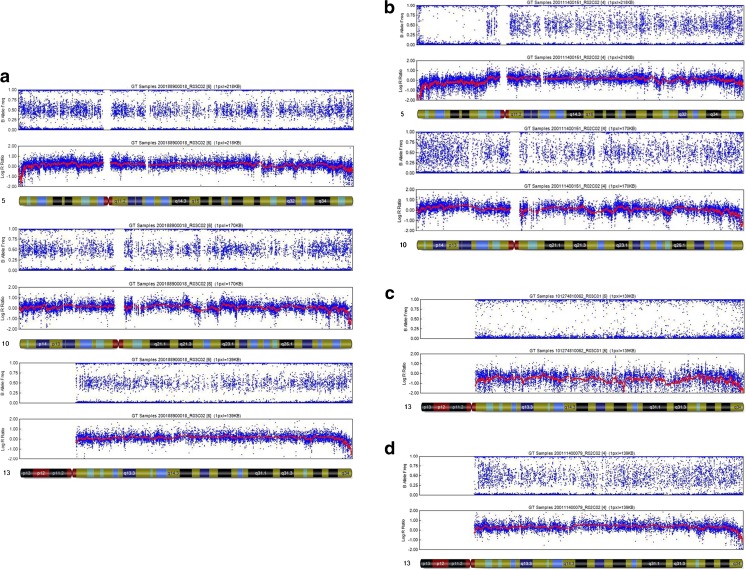
Normal and abnormal molecular karyotypes of trophectoderm cells (TE) from blastocysts biopsied using MDA-based SNP microarray. **a** Normal diploid diagnostic reading obtained from TE for Chromosomes (Chr.) 5, 10, and 13. **b** Deletion of p13.1 → pter reading of Chr. 5 combined with a duplication of p12.1 → pter reading of Chr. 10, which came from the same blastocyst of a 46,XY, t(5;10)(P15;P12) carrier couple. AA, AB, and BB alleles were observed in p13.1 → qter of Chr. 5 and from p12.1 → qter of Chr. 10. However, AA and BB alleles were detected without AB in p13.1 → pter of Chr. 5, along with AAA, AAB, and BBB from p12.1 → pter of Chr. 10. A significant shift in the smooth log R ratio is shown in p13.1 → pter of Chr. 5 and p12.1 → pter of Chr. 10. **c** Monosomy reading of Chr. 13. AA and BB alleles are illustrated without AB alleles represented. A significant shift in the smooth log R ratio was detected, consistent with the monosomy karyotype. **d** Trisomy reading of Chr. 13. AAA, AAB, ABB, and BBB are shown without the AB alleles represented. A significant shift in the smooth log R ratio was observed, consistent with the trisomy karyotype

Examples of normal and abnormal molecular karyotypes are shown in Fig. 2.

## Discussion

Patients who carry balanced translocations have a high incidence of abnormal meiotic segregations, which decreases the chance of finding an embryo for transfer in carriers. This is exacerbated when carriers also have a poor ovarian reserve. It is critical for the clinic to determine a threshold of the number of blastocysts available to biopsy to maximize the chance of obtaining at least one transferrable blastocyst.

In this study, rcp carriers had a significantly higher rate of unbalanced embryos compared with RT carriers. For rcp carriers, only an alternate meiotic segregation pattern can lead to the production of chromosomally normal/balanced gametes, which only accounts for 1/9. For RT patients, the rate of obtaining alternate embryos derived from alternate division is 1/3. The data in the study of Chang et al. of 31.3 % transferrable embryos from 514 biopsied blastomeres was similar to that [12].

However, the normal/balanced rates of 42.2 % in RT and 26.9 % in rcp were both higher than genetically expected in this study. A recent study by Idowu et al. including 539 biopsied embryos used for the same CytoSNP-12 arrays also showed up to 37 % of RT embryos and 19 % of rcp embryos were euploid [13]. The reason for a lower transferrable rate in their study might be attributed to two factors. First, nearly half of the embryos (202/539, 37.5 %) were from women ≥35 years compared to 24/182 (13.2 %) in our study. Second, only one fifth of embryos (102/539, 18.9 %) was biopsied on D5, and the remaining embryos were biopsied at the cleavage stage. In our study, only trophectoderm biopsy was carried out, and the proportions of D5 and D6 blastocysts biopsied were 957/1432 (66.8 %) and 475/1432 (33.2 %), respectively.

The adverse relationship between advanced maternal age and embryo euploidy has been universally accepted [14]. According to our statistical inequality, only when the conditions of female age younger than 37 years with basal FSH levels under 11.4 IU/L were the conclusions from ROC analysis credible.

The aneuploidy rate was also inversely associated with development stage. Many studies recommend blastocyst-stage biopsy rather than cleavage-stage biopsy, which has the advantage of reduced biopsy-related damage, lower mosaic rate, and higher pregnancy rate [15–17]. A previous study reported more than half of cleavage stage embryos (57.7 % of 134 single blastomeres) exhibited mosaicism [18]. Another study evaluated 280 embryos from rcp carriers (56 blastocysts and 224 cleavage-stage embryos) and observed that 57.5 % were aneuploid with or without the unbalanced parental translocation. When biopsy only included trophectoderm cells from blastocysts, the aneuploidy prevalence dropped to 26.7 % [19], which was similar to the 26.4 % in the rcp group in the current study. The progression of embryos developing from the cleavage stage to the blastocyst stage, referring to blastulation, is clinically important. Because most unbalanced embryos in the cleavage stage degenerated or stopped developing because of chromosomal abnormalities, they did not achieve complete blastulation. Therefore, the finding that the proportion of transferrable embryos tended to increase from the cleavage stage to the blastocyst stage might aid our understanding of this phenomenon. Of note, both D5 and D6 blastocysts were recruited in our study. According to previous studies, embryos that were slower to develop presented with a higher proportion of chromosomal abnormalities compared with embryos that progressed normally [20–22]. Therefore, in our study, we verified that the proportion of normal/balanced blastocysts in D5 blastocysts was significantly higher (318/908) than that in D6 blastocysts (125/433) (*P* = 0.025). Therefore, if the proportion of D5 blastocysts was overwhelming, one normal/balanced blastocyst might be obtained from less biopsied blastocysts.

In the present study, the number of biopsy blastocysts was correlated to transferrable outcomes in a cohort of female patients aged younger than 37 years and with a basal FSH level less than 11.4 IU/L. This study concluded that one normal/balanced embryo was likely to be obtained from 2 blastocysts in the RT group and 4 blastocysts in the rcp group. Median values of biopsied blastocysts (both D5 and D6 recruited) and normal/balanced embryos from a published research study based on SNP were presented as 4, 2 in the RT group and 4, and 1 in the rcp group [23]. This indicated that RT carriers were two times more likely to have a chance of a normal/balanced embryo than rcp carriers. Indeed, a majority of RT carriers (94.1 %) and more than half of rcp carriers (65.6 %) in their first COS cycle had blastocyst numbers higher than the threshold calculated here. As for these couples, our findings could be used to predict how many normal/balanced embryos could be obtained if the blastocysts qualifying for biopsy reached a certain number.

This study also demonstrated that 5.9 % of RT couples and 34.4 % of rcp patients, diagnosed as having a diminished ovarian reserve or poor response, did not obtain the desired number of blastocysts in their first cycle. In these cases, what clinical strategy should be followed? We share the same opinion with Tulay et al. and Chatziparasidou A et al. [24, 25] who suggested that serial cycles of ovarian stimulation should be undertaken. According to the study of Scriven et al., when each additional embryo was available for biopsy, there was a 13 % higher opportunity of a live birth [26].

To avoid mistakes and cross-contaminations during handling, each SNP chip was only used once for each couple regardless of how many embryos were analyzed in our center. The price of one chip is estimated at US$3200 in China. If a biopsy is routinely requested after every COS cycle for patients with limited blastocysts, there is likely to be unnecessary expense before obtaining a transferrable blastocyst. In addition, the current policy of China does not allow charging on a per embryo basis. Thus, testing embryos after serial cycles of ovarian stimulation is recommended for couples with limited numbers of blastocysts.

One main limitation of this study was the preprocessing techniques of the SNP chips used for diagnosis. SNP chips avoid many drawbacks inherent in array CGH (which uses ratio labeling), resulting in a lower density and an inability to identify some karyotypes (69, XXX appeared the same as 46, XX) [27]. However, all current techniques have the same limitation, and therefore, it is difficult to distinguish a balanced embryo from a normal embryo unless using parental guidance haplotype analysis. In addition, due to the detection capability of SNP chips, only segmental aneuploidy of no less than 5 megabases (Mb) can be distinguished from the vast majority of unbalanced segregations. A specific abnormal product would not be scored if small copy number of variants or polymorphisms were under this cutoff value. The mean size of the chromosomal unbalance detected was up to 49 Mb for duplication errors and 35 Mb for deletion errors [28].

Another limitation was the use of MDA, which has a relatively high preferential amplification and allele dropout (ADO) rate (average of 25 %) [29], particularly in regions close to centromeres and telomeres [30]. Furthermore, some specific chromosomal regions, such as 1q42, 4q35, and 6p25, were reported to show a loss of representation after MDA [31]. All these detrimental factors might confound technicians when making a diagnosis. However, outcomes from studies have shown these disadvantages do not have a determinative role. In our center, the preferential use of nanoliter reactors and analysis of multiple loci (STRs, short tandem repeats) might help to ameliorate amplification bias [29, 32]. The failure rate was decreased to 0.002 % when three STRs markers were placed either side of the translocation breakpoints [33]. Furthermore, this platform provided an authentic diagnosis in other PGD centers, similar to that in our center [13, 18, 34].

## Conclusion

To increase the likelihood of obtaining at least one normal/balanced embryo following PGD by SNP microarray, we recommend that for females aged younger than 37 years with a basal FSH level under 11.4 IU/L, the number of blastocysts biopsied should be no less than 2 for RT carriers and no less than 4 for rcp. The accumulation of embryos before biopsy is a feasible strategy for patients with a limited number of embryos available for PGD. The outcome of our study might be more persuasive if more cases with a single transferrable embryo after diagnosis were available for analysis.
